# Molecular Evolution of the Archaeal DNA-Dependent RNA Polymerase: Cooperative Changes in Subunit Composition and Specific Domains of Small Subunits

**DOI:** 10.3390/ijms27114679

**Published:** 2026-05-22

**Authors:** Elena K. Shematorova, George V. Shpakovski

**Affiliations:** National Research Centre “Kurchatov Institute”, Moscow 123182, Russia; shematorova_ek@nrcki.ru

**Keywords:** archaeal RNA polymerase, Rpo8 subunit, Rpo6 subunit, domain structure, cooperative changes of small subunits, transcripton factors TFB and TFE, evolution of multi-subunit RNA polymerases

## Abstract

The subunit composition and tertiary structure of DNA-dependent RNA polymerases in archaea, bacteria, and eukaryotes are currently well understood. The single RNA polymerase of archaea resembles the nuclear RNA polymerase II of eukaryotes in its composition and consists of 10–12 subunits. Perhaps the only exception that seems to confirm this rule is the Rpo8 subunit (homologue of the eukaryotic Rpb8), which only some classes of archaea have. The development of metagenomic sequencing has led to a significant revision of the classification system of prokaryotes, in particular to the identification of a number of new Archaea evolutionary lineages. This makes it possible to analyze the subunit composition and structure of RNA polymerase of all currently isolated archaeal phyla. Our analysis shows that the Rpo8 subunit is present only in the RNA polymerase of Archaea species from the Thermoproteota of the Thermoproteati superphylum and from the whole superphylum Promethearchaeati, formerly known as the Asgard. After analyzing the changes in the small Rpo6 subunit (homologue of eukaryotic Rpb6), functionally interacting with Rpo8, we noticed that the largest number of changes in the primary and domain structures of this small subunit occurred in archaeal phyla that lack Rpo8. Shortened forms of Rpo6 without N- or C-terminal regions were observed only in representatives of archaea with an RNA polymerase that does not contain the Rpo8 subunit. Our analysis shows that the changes in Rpo6 are an adaptation of a multisubunit transcription complex to the disappearance of Rpo8. Most likely, the Rpo8 subunit was present in the RNA polymerase of the Last Common Ancestor of Archaea (LCAA) and, in the course of evolution, disappeared in the superphyla Euryarchaeota and Nanobdellati and two divisions of the Thermoproteati superphylum: Bathyarchaeota and Thaumarchaeota.

## 1. Introduction

DNA-dependent RNA polymerases are enzymes that catalyze the complementary synthesis of RNA using one of the DNA strands as a template. These enzymes play a crucial role in the transcription process, which is the first step in gene expression. All multisubunit RNA polymerases responsible for the transcription of a cell’s genetic material have a common structure and function through a similar molecular mechanism, suggesting that they all evolved from the Last Universal Common Ancestor (LUCA) [1].

While archaea and eubacteria use a single type of RNA polymerase to transcribe all of their genes, eukaryotes have three main nuclear RNA polymerases that specialize in transcribing separate, independent sets of genes. The two largest RNA polymerase subunits, β’ and β subunits in bacteria, Rpa1 and Rpa2, Rpb1 and Rpb2, Rpc1 and Rpc2 in eukaryotic nuclear RNA polymerases I-III, respectively, and Rpo1 and Rpo2 in archaea, originate from a common ancestor and contain double-ψ β-barrel motifs that form the enzyme’s active center and participate in the binding of the DNA template and the nascent RNA chain [1].

Two motifs in the primary structures of the Rpc40 and Rpc19 subunits of RNA polymerases I and III, Rpb3 and Rpb11 of RNA polymerase II, and Rpo3 and Rpo11 of archaea are similar to the N-terminal domain of the α subunit of eubacteria. The Rpc40 and Rpc19 subunits, as well as Rpb3 and Rpb11, form stable heterodimers in vivo [2,3,4,5]. The α subunit forms a homodimer and is involved in the assembly of bacterial RNA polymerase and the activation of transcription [6,7]. The C-terminal part of the α subunit is important for ρ-dependent terminal RNA processing in *E. coli* [8].

It was shown that yeast Rpb3 can interact with Rpb2 and further with Rpb1 [9]. In RNA polymerase from archaea, the specific ferredoxin domain of the Rpo3 subunit also affects the assembly of the enzyme: when this domain was completely or partially removed in *Methanosarcina acetivorans*, the interaction of the Rpo3-Rpo11 heterodimer with the catalytic subunits Rpo2 and Rpo1 was significantly weakened [10].

The Rpb3-Rpb11 heterodimer provides interaction of RNA polymerase II with the Mediator complex [11], transcription factors ATF4 [12,13], AATF (Che1) [14], and MyoD [15]. The Rpb3-Rpb11 subcomplex also plays an important role in the termination of small RNA transcription in yeast [16].

The human heterodimer is tolerant to significant changes in the C-terminal fragment of Rpb11, which allows two variants of the human Rpb11 subunit [17,18] to dimerize with Rpb3 with approximately equal efficiency [19].

**Rpb10 and Rpb12.** Rpb10 and Rpb12 of eukaryotes are homologous to the Rpo10 and Rpo12 subunits of archaea. Rpo10 and Rpo12, as well as Rpb10 and Rpb12, fill in the grooves in the second largest subunit of RNA polymerase (Rpo2 and Rpb2, respectively). They act as structural adapters between Rpo2 and Rpo3 or Rpb2 and Rpb3, respectively [3,20]. However, Rpb10 and Rpb12 have some specific functions in all three classes of eukaryotic RNA polymerases [3,21,22,23,24].

Rpb10 subunit hybrids with the Rpo10 subunit of archaea are functional in yeast, indicating that this protein plays a similar role in organizing corresponding transcription complexes in archaea and eukaryotes [24]. Similarly, the *Methanosarcina mazei* Rpo12 subunit and the eukaryotic polymerase Rpb12 subunit are interchangeable in vivo and in vitro [25]. Additionally, the Rpo12 homolog in archaea plays a role in transcription initiation by facilitating the melting of the DNA template and stabilizing the open complex [20].

**Rpb5.** The Rpb5 subunit forms many contacts with the C-terminus of the largest RNA polymerase subunit. Rpo5 is involved in DNA melting and the early stages of transcription initiation [26,27,28], and it also interacts with TFE [29]. The N-terminal domain of the eukaryotic subunit Rpb5, which is approximately 2/3 of the total length of the protein polypeptide chain in terms of its primary structure, interacts with the hepatitis B virus transactivator X protein HBx [30], TIP-120 [31], TAFII68 [32], TFIIB [33], and the RAP30 subunit of the TFIIF factor [34].

**Rpb6**. The Rpb6 subunit has a homolog in archaea, Rpo6, and is structurally and functionally similar to the ω subunit in eubacteria [35]. This subunit is required for the assembly of at least RNA polymerases I and II, as well as for the stability of the largest subunits of these two enzymes [36]. The addition of recombinant Rpb6 subunit in vitro to the inactive form of *S. cerevisiae* RNA polymerase I leads to partial restoration of its transcriptional activity, indicating the involvement of Rpb6 in the formation of the structural integrity and functional activity of RNA polymerase I [37].

Rpb6 is one of the most conserved small subunits of eukaryotic RNA polymerases. Replacing the Rpb6 subunit in *S. cerevisiae* cells with homologs from *Schizosaccharomyces pombe* and *Homo sapiens* results in the formation of viable yeast cells, indicating the functional conservation of Rpb6 in eukaryotes [38,39,40]. The Rpb6 subunit plays an important role in the binding of the Rpb4-Rpb7 subcomplex to RNA polymerase II in eukaryotes [41]. The N-terminal region of Rpb6 interacts with the PH domain of the p62 subunit of the general transcription factor TFIIH [42].

**Rpb8.** The determination of the spatial structure of the Rpb8 subunit in solution allowed this protein to be classified as an OB family (oligosaccharide and oligonucleotide binding proteins) and suggested that Rpb8 binds single-stranded oligonucleotides [43,44]. In archaea, the Rpo8 protein was first discovered among the subunits of the RNA polymerase *Sulfolobus acidocaldarius* as the G subunit [45,46] and is present only in some species of archaea [47]. The main partner of Rpb8 in the RNA polymerase II complex is the largest subunit, Rpb1 [2]. Contacts with Rpb1 mainly occur in the Pore 1 domain. Genetic studies have also revealed a functional interaction between Rpb8 and the Rpb6 subunit [48]. It appears that Rpb6, Rpb8, and the Pore1 module form a functionally integrated domain of RNA polymerase II.

**The Rpb7–Rpb4 subcomplex.** The most notable difference between the RNA polymerase of archaea and eukaryotes and the bacterial transcription enzyme is the “stalk”, which is located at the base of the enzyme and includes the Rpo4 and Rpo7 subunits in archaea and the Rpb4 and Rpb7 subunits in eukaryotes, respectively. The two subunits form a stable complex that binds reversibly to RNA polymerase II in *S. cerevisiae* [49], but is irreversibly incorporated into RNA polymerase in archaea [50]. It performs multiple functions in the transcription cycle: it promotes the formation of open complexes during transcription initiation and facilitates the action of the basal transcription factor TFE [51,52,53,54]. During elongation, Rpo4-Rpo7 and Rpb4-Rpb7 increase the processivity of the corresponding RNA polymerases, and in archaea, Rpo4-Rpo7 also contribute to transcription termination [55].

**Subunits with specific domains.** Rpb9 is the only subunit found exclusively in eukaryotic RNA polymerase. Rpb9 affects the interaction of RNA polymerase II with the basal factor TFIIF and, therefore, the selection of the transcription start site, as well as the attachment of the elongation factor TFIIS and the speed and accuracy of transcription [56,57]. Rpo13 is the only archaeal specific subunit found in some genomes of the class Thermoprotei. In the three-dimensional structure of the *Sulfolobus shibatae* RNA polymerase, it is located in the region of the N-terminal part of Rpo5 and interacts with double-stranded DNA, providing additional fixation of the RNA polymerase in the promoter region [58].

## 2. Results

### 2.1. Domain Composition and Tertiary Structure Study of the Rpo8 Subunit of Archaea

**Figure 1:**
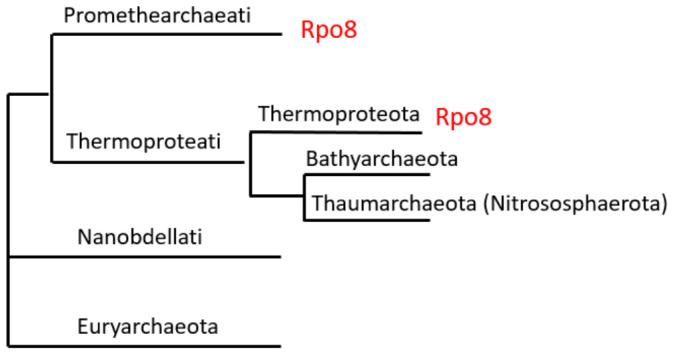
Archaeal taxonomy according to work [61]. The presence of the Rpo8 subunit in the archaeal taxa is marked in red.

It has been previously shown that only members of the Thermoproteota have the Rpo8 subunit in their RNA polymerase [47]. At the same time, Bathyarchaeota and Thaumarchaeota, two branches of archaea that diverged from Thermoproteota, lack the Rpo8 subunit in their RNA polymerase (Figure 1). The discovery of the first members of the new phylum Asgardarchaeota (recently renamed Promethearchaeati [59]) has revealed that their genomes also contain a gene encoding the Rpo8 subunit. The Rpo8 gene has been found in almost all Promethearchaeati species whose genomes have been completely sequenced [60].

**Figure 2:**
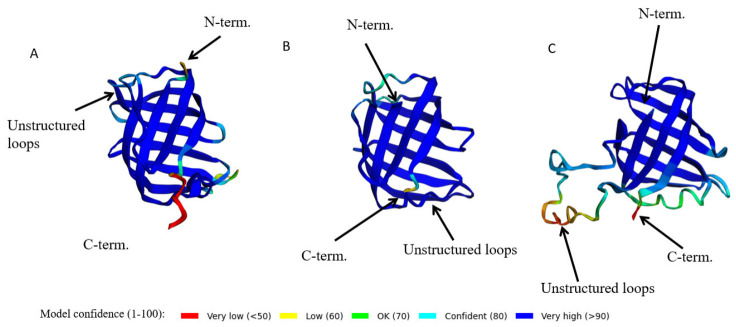
Three-dimensional structure of Rpo8 predicted by AlphaFold3: (**A**) sp|Q980L5.1|RPO8 [*Saccharolobus solfataricus* P2]; (**B**) MEX2705314.1 [Candidatus *Freyrarchaeum guaymaensis*]; (**C**) NP_014867.1 [*Saccharomyces cerevisiae* S288C].

The primary structure of the Rpo8 protein is very variable: when comparing representatives of the same class, the maximum percentage of identical amino acids can be as low as 28–30% in Thermoproteota and 37–40% in Promethearchaeati. The most conserved amino acids are those involved in the formation of the tertiary structure of this RNA polymerase subunit [48]. When comparing the primary structures of the Rpo8 subunit in representatives of the Promethearchaeoti and Thermoproteota groups, the PSI-BLAST program (online version) does not detect homology between them in most cases. A comparison of the Rpo8 of representatives of the Promethearchaeoti group with the Rpb8 subunit of various eukaryotes shows that their amino acid sequences have similarity of about 22–35%. The three-dimensional structures of the Rpo8 protein of various representatives of Thermoproteota and Promethearchaeati are very similar. The 3D structures of the eukaryotic Rpb8 subunit differ from those of archaeal proteins only in the presence of long unstructured loops located between the conserved elements of the tertiary structure (Figure 2).

The tertiary structure of the eukaryotic Rpb8 subunit consists of a specific oligonucleotide/oligosaccharide-binding domain with a large number of exposed basic, aromatic, and hydrophobic residues located on β-strands and loops [43,44]. The main function of this subunit is believed to be the binding of the growing transcript that exits the active site along groove 2 [2,16]. The interaction of RNA with Rpo8 in archaea can affect the upper part of the Pore 1 module of the Rpo1 which is located directly below Rpo8. It is through this Pore 1 module of the largest subunit of RNA polymerase that the Rpo8 subunit in archaea can affect the active site of the enzyme, initiating transcription termination. Since this does not require additional factors, we assume that this is the most ancient mechanism of transcription termination.

Next to Rpo8 in the three-dimensional structures of RNA polymerase from *Saccharolobus solfataricus* (PDB ID: 3HKZ) [62] and *Sulfolobus shibatae* (PDB ID: 2WAQ) [58] is the domain III of the Rpo3 subunit, containing a cluster of ferredoxins [62], which is also capable of RNA binding [63]. This could indicate that the functions of these structures are somewhat duplicated. This explains the disappearance of Rpo8 from the RNA polymerase of most archaea, as it allows for a slightly simpler assembly of the enzyme, which requires more energy and time compared to the RNA polymerase of eubacteria. In yeast nuclear RNA polymerases, the Rpb8 subunit functionally interacts with Rpb6 [48], so the loss of the Rpo8 subunit in some groups of archaea should have led to compensatory structural changes in the small Rpo6 subunit of RNA polymerase, which may play an important role in adaptation of these microorganisms to new conditions and habitats.

Rpo8 is present in only two archaea taxa: Thermoproteota (Thermoproteati) and Promethearchaeati (Figure 1). A comparison of the primary structures of Rpo8 showed a low degree of homology in both Thermoproteota and Promethearchaeati phyla. At the same time, the fact that only the Rpo8 protein from the Promethearchaeati has a detectable homology with the Rpb8 subunit of eukaryotes indicates that the Thermoproteati and the Promethearchaeati are two separate branches of Archaea that diverged a long time ago. This is also a strong additional argument in favor of the hypothesis that it was prokaryotic organisms related to the Promethearchaeati that played the role of the ancestor of eukaryotic cells [59,60].

### 2.2. Study of the Domain Composition and Tertiary Structure of the Rpo6 Subunit of Archaea

In this section, we will consider the primary and tertiary structures of Rpo6 of all currently known archaea species [61].

#### 2.2.1. Thermoproteati

##### Thermoproteota

**Figure 3:**
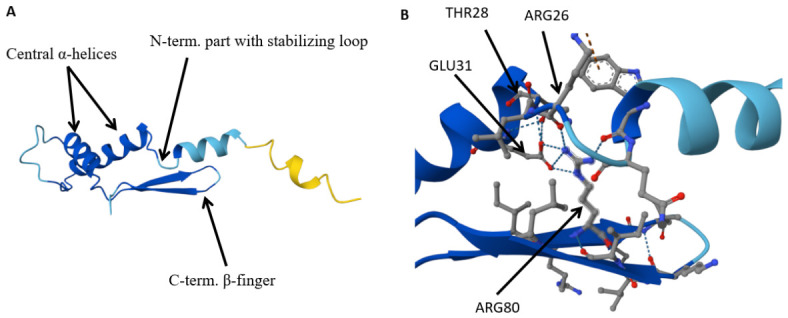
Three-dimensional structure of Rpo6 predicted by AlphaFold3: (**A**) (basic form)—WP_009992332.1 [*Saccharolobus solfataricus*]; (**B**) Interaction between stabilizing loop and C-terminal β-finger of Rpo6 (*Saccharolobus solfataricus*).

Representatives of Thermoproteota include thermophiles and hyperthermophiles, as well as inhabitants of marine environments, soil and freshwater. In the three-dimensional structure of *S. solfataricus* RNA polymerase, Rpo6 interacts tightly with the N- and C-terminal domains of the Rpo5 subunit [62], forming a single platform for interaction with the archaeal transcription initiation factor TFE [64,65]. All members of Thermoproteota have a basic form with an N-terminal sequence containing a stabilizing loop and a C-terminal β-finger consisting of two antiparallel β-strands (Figure 3A). The stabilizing loop consists of 1–2 charged amino acid residues, which provide interaction with the C-terminal β-finger (Figure 3B). All members of Thermoproteota have the Rpo8 subunit in their RNA polymerase and only one form of the Rpo6 subunit, with a length of 75–120 amino acids (Table 1).

##### Bathyarchaeota

**Figure 4:**
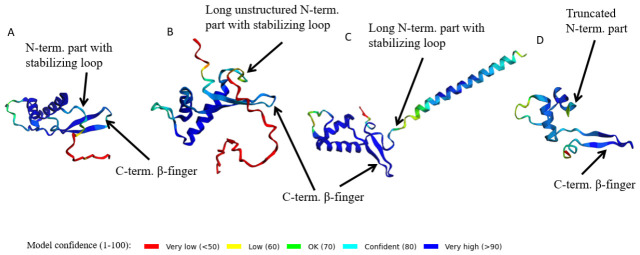
Three-dimensional structure of Rpo6 predicted by AlphaFold3: (**A**) (Rpo6, form 1, basic)—MCS7120306.1 [Candidatus Bathyarchaeota archaeon]; (**B**) (Rpo6, form 2)—TFH14440.1 [Candidatus Bathyarchaeota archaeon]; (**C**) (Rpo6, form 3)—MEM2102539.1 [Candidatus Bathyarchaeia archaeon]; (**D**) (Rpo6, form 4)—MBS7632940.1 [Candidatus Bathyarchaeota archaeon].

Bathyarchaeota are widespread archaea abundant in nutrient-poor bottom sediments. They are evolutionarily close to Thermoproteota, but the RNA polymerase of Bathyarchaeota does not contain the Rpo8 subunit. We identified four different forms of Rpo6 in different Bathyarchaeota species, and their tertiary structure was predicted using the AlphaFold3 program. The first form is a basic Rpo6 protein similar to Rpo6 Thermoproteota. The second form has an elongated N-terminal domain containing a long unstructured sequence (30–100 amino acid residues) rich in charged amino acids. In the primary structure of the third form, regions with charged amino acids in the elongated N-terminal part of Rpo6 may include α-helices of varying lengths. When comparing the primary sequences of the N-terminal regions of Rpo6 in different members of the Bathyarchaeota, no significant homology was found. Similarly, a comparison of the primary sequences of the extended N-terminal forms of Rpo6 in Bathyarchaeota and the Rpb6 subunit in eukaryotes did not reveal significant homology. The fourth form of Rpo6 is a shortened form that does not contain the N-terminal sequence and part of the central α-helix. The appearance of elongated and shortened forms in Bathyarchaeota appears to be related to the absence of the Rpo8 subunit, which leads to changes in the three-dimensional structure of the RNA polymerase (Figure 4).

##### Thaumarchaeota

**Figure 5:**
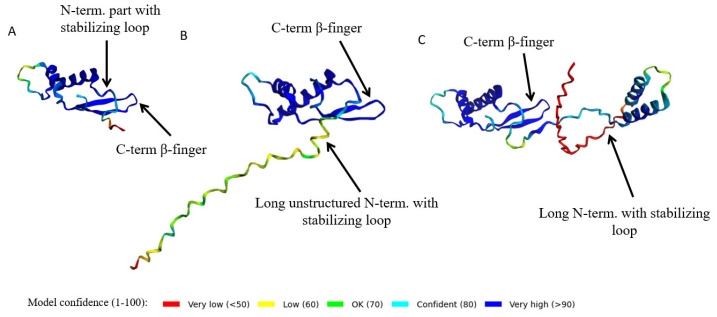
Three-dimensional structure of Rpo6 (Thaumarchaeota) predicted by AlphaFold3: (**A**) (Rpo6, form 1)—MDR0318834.1 [Nitrososphaerota archaeon]; (**B**) (Rpo6, form 2)—MDG6996227.1 [Nitrososphaerota archaeon]; (**C**) (Rpo6, form 3)—NWK04602.1 [Thaumarchaeote KM3_126_D02].

Thaumarchaeota is a large and diverse group of archaea that inhabit various marine, freshwater, terrestrial, and extreme ecosystems. Most of the identified organisms in this phylum are ammonia oxidizers and may play important roles in biogeochemical cycles such as the nitrogen and carbon cycles. Bathyarchaeota and Thaumarchaeota are evolutionarily related phyla of archaea. Three different forms of Rpo6 have been found in Thaumarchaeota (Figure 5).

The first form is the basic Rpo6 protein. The second and the third Rpo6 forms are similar to Bathyarchaeota ones (Figure 5). The N-terminal extended Rpo6 sequences in Thaumarchaeota are unique and do not share similarities with the N-terminal sequences of Rpo6 in archaea and eukaryotes.

**Figure 6:**
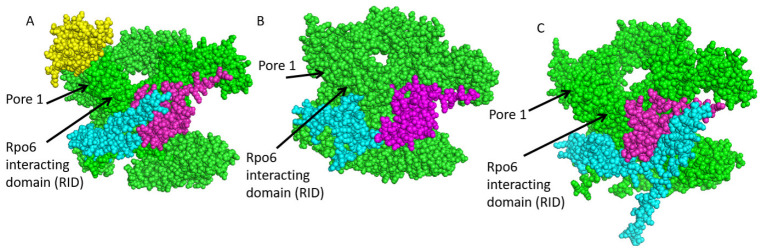
Three-dimensional interaction model of Rpo5 (magenta), Rpo6 (cyan), Rpo8 (yellow) and the largest RNA polymerase subunit Rpo1 (green) predicted by AlphaFold3. (**A**) Thermoproteota (*Saccharolobus solfataricus* (Rpo 8: WP_218258916.1; Rpo5: WP_012711883.1; Rpo6: WP_009992332.1; Rpo1: WP_218258963.1, WP_009990475.1); (**B**) Bathyarchaeota (Rpo5: MEM2791772.1; Rpo6: MEM2791895.1; Rpo1: MEM2791770.1); (**C**) Thaumarchaeota (Rpo5: NWK05067.1; Rpo6: NWK04602.1; Rpo1: NWK05069.1). The confidence score of these models in all structured regions of the archaeal RNA polymerase is 70–90%.

As shown in Figure 6, Rpo6 interacts with Rpo5 and the Rpo6-interacting domain (RID) of Rpo1 [2]. Rpo5 also interacts with the RID [62]. These three components form a compact region in the three-dimensional structure of the RNA polymerase, where each element interacts with two others (Figure 6A). The disappearance of Rpo8 leads to a modification of the Pore 1 domain (one of the most variable in archaea—see Table 2), which in turn affects the adjacent and interacting RID. Apparently, the Rpo6-RID-Rpo5 region cannot be modified only by changing the primary structure of these sequences; therefore, it undergoes modifications by elongating (or shortening) Rpo6 and changing the contacts between its components. Extended variants of Rpo6 form additional interactions with RID (Figure 6B) or with Rpo5 (Figure 6C), stabilizing the three-dimensional structure of the RNA polymerase in this region in response to changes in the structure of the Pore 1 domain.

#### 2.2.2. Promethearchaeati

**Figure 7:**
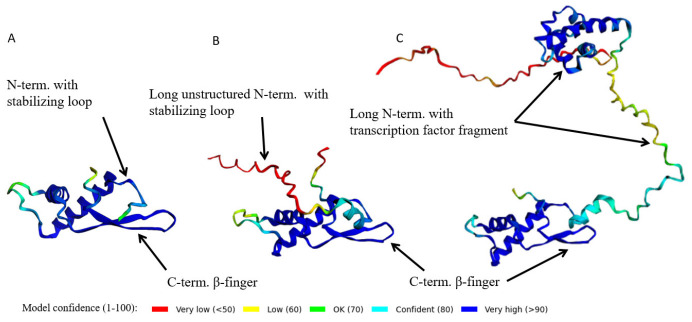
Three-dimensional structure of Rpb6 (Promethearchaeati) predicted by AlphaFold3: (**A**) MEX2705833.1 [Candidatus *Freyrarchaeum guaymaensis*]; (**B**) HKZ40234.1 [Candidatus Hodarchaeales archaeon]; (**C**) MHA1137641.1 [Candidatus Thorarchaeota archaeon].

A recently discovered superphylum of archaea with many proteins similar to eukaryotes, Promethearchaeati are widely distributed in seawater, marine sediments, and are also found in animal bodies, plant rhizosphere, unsalted sediments and soils, and freshwater. Two different forms of Rpo6 have been found in Promethearchaeati, and their tertiary structure has been predicted using the AlphaFold3 program. The first form is a basic Rpo6. The second form has an extended N-terminal domain with an unstructured sequence (Figure 7). Typically, the N-terminal sequences of Promethearchaeati contain many positively and negatively charged amino acids. An interesting feature of the Rpo6 of Thorarchaeota is the presence of a long N-terminal sequence in which the AlphaFold3 program detected a helix–loop–helix transcription factor fragment [66].

It may seem surprising that the structure of the RNA polymerase of Promethearchaeati combines the presence of Rpo8 and elongated forms of Rpo6, which are practically not found in Thermoproteota. At the same time, the primary structure of Rpo8 in Promethearchaeati differs significantly from that of Rpo8 in Thermoproteota, which leads to changes in the Pore 1 domain and compensatory elongation of Rpo6. Until recently, it was believed that Rpo6 in archaea corresponds to the C-terminal part of Rpb6 in eukaryotes. As we can see, representatives of the Promethearchaeati (as well as Thaumarchaeota and Bathyarchaeota) have long isoforms with a structural organization similar to that of the full-length Rpb6 subunit.

**Figure 8:**
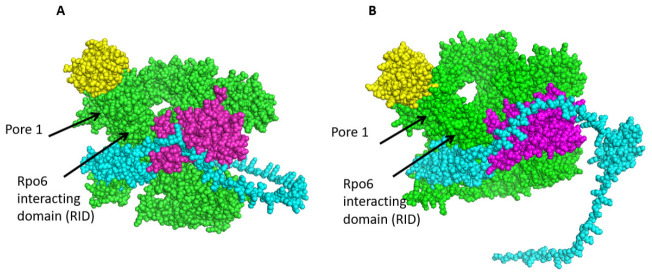
Spatial model of interactions of Rpo5 (magenta), Rpo6 (cyan), and Rpo8 (yellow) with the largest subunit of RNA polymerase Rpo1 (green) Promethearchaeati predicted by AlphaFold3. (**A**) Candidatus Helarchaeota archaeon (Rpo8: MHA1266732.1; Rpo5: MHA1263799.1; Rpo6: MHA1266008.1; Rpo1: MHA1266734.1; (**B**) Candidatus Thorarchaeota archaeon (Rpo8: MHA1136359.1; Rpo5: MHA1138257.1; Rpo6: MHA1137641.1; Rpo1: MHA1137817.1, MHA1137816.1). The confidence score of these models in all structured regions of the archaeal RNA polymerase is 70–90%.

The interaction models between the Rpo1, Rpo5, Rpo6, and Rpo8 subunits in the Promethearchaeati superphylum show additional Rpo6-Rpo5 interactions in Helarchaeota or Rpo6-Rpo5 and Rpo6-RID interactions in Thorarchaeota. The N-terminal domain of Rpo6, enriched in charged amino acid residues, enables binding additional transcription factors in Helarchaeota and/or to stimulate transcription using a transcription factor fragment in Thorarchaeota (Figure 8).

#### 2.2.3. Euryarchaeota

Representatives of the Euryarchaeota superphylum are diverse in appearance and metabolic properties. This evolutionary branch of archaea includes organisms with different lifestyles, including methanogens, halophiles, and sulfate reducers. Extreme thermophiles are found in all classes.

**Figure 9:**
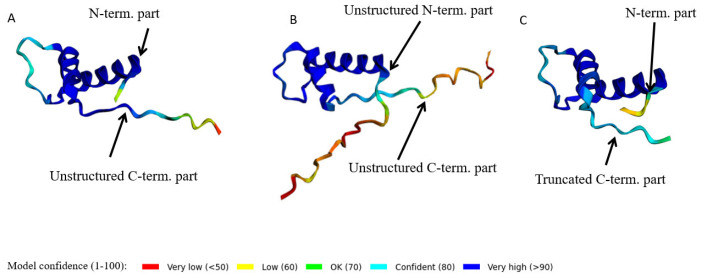
Three-dimensional structure of Rpo6 (Euryarchaeota) predicted by AlphaFold3: (**A**) WP_010917483.1 [*Thermoplasma volcanium*]; (**B**) WP_048098288.1 [*Archaeoglobus sulfaticallidus*]; (**C**) EGQ39947.1 [Candidatus *Nanosalinarum* sp. J07AB56].

Two forms of Rpo6 have been found in the genomes of Euryarchaeota. In the first form, there is no stabilizing loop in the N-terminal region. The C-terminal β-finger is also missing; its three-dimensional structure is disrupted due to point mutations or short deletions. In the second form of Rpo6, only a short, unstructured N-terminal sequence and the central part of the protein, consisting of two α-helices, were preserved. Thus, representative members of this superphylum, along with Nanobdellati, have the shortest primary structure of the Rpo6 protein (52–55 amino acids) (Figure 9).

The three-dimensional structure of two representatives of Euryarchaeota, *Thermococcus kodakarensis* (PDB ID: 4QIW) [67] *and Pyrococcus furiosus* (PDB ID: 8CRO) [68], has been determined using X-ray diffraction and cryo-electron microscopy, respectively. The structures of RNA polymerases show that the Rpo5 and Rpo6 subunits occupy a similar position as in *S. solfataricus*, but do not touch each other. They are located at some distance from each other due to the absence of the C-terminal domain in Rpo6. Such a radical change in the primary structure of Rpo6 in Euryarchaeota species should have provided significant advantages for the vital activity of these microorganisms. Apparently, the disruption of the interaction between Rpo5 and Rpo6 limits the binding area of the TFE initiation factor by the Rpo6 and Rpo7 subunits, excluding Rpo5. Indeed, it has been shown that in *T. kodakarensis*, Rpo5 is not involved in TFE binding [53].

#### 2.2.4. Nanobdellati

Nanobdellati is a superphylum of archaea that was first described in 2013. Nanobdellati cells are the smallest among all archaea and are measured in nanometers. Additionally, these archaea are characterized by a very small genome (0.5–1.1 MB) and a symbiotic lifestyle with other archaea and even bacteria. Their existence as symbionts seems to have led to an increased rate of mutations in these organisms (the need to coevolve with the symbiont), the loss of a number of genes, and the exchange of genes with their hosts. All of this makes it difficult to establish the origin and classification of the Nanobdellati superphylum [69]. The increased level of mutations is the cause of a phylogenetic artifact known as long-branch attraction (LBA), which causes branches of the phylogenetic tree that are not closely related to be grouped together [70]. As a result, the monophyly and exact placement of the Nanobdellati superphylum on the evolutionary tree are controversial [71,72,73,74,75].

**Figure 10:**
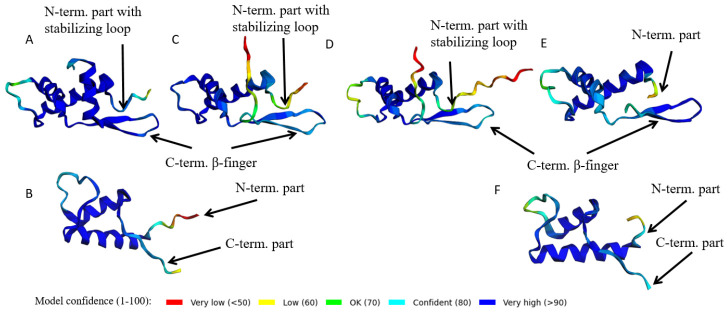
Three-dimensional structure of Rpo6 (Nanobdellati) predicted by AlphaFold3: (**A**) NCO97327.1 [Candidatus Aenigmarchaeota archaeon]; (**B**) HDD46341.1 [Candidatus Aenigmarchaeota archaeon]; (**C**) MDP7080753.1 [Candidatus Undinarchaeales archaeon]; (**D**) HIJ98900.1 [Candidatus Naiadarchaeales archaeon SRR2090153.bin1042]; (**E**) MDD3159423.1 [Candidatus *Ainarchaeum* sp.]; (**F**) MCD6523013. [Candidatus Iainarchaeota archaeon].

We analyzed the domain composition and three-dimensional structure of the Rpo6 RNA polymerase subunit of this superphylum. Aenigmarchaeota have two forms of Rpo6: a basic form and a shortened form that lacks the C-terminal domain, similar to the form found in Euryarchaeota. Undinaarchaeota and Naiadarchaeota also have basic forms (Figure 10).

**Figure 11:**
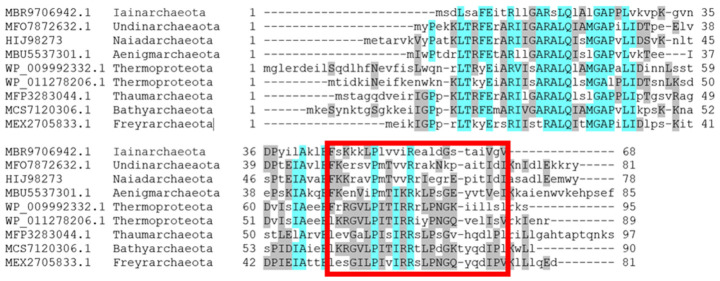
Multiple alignment of the Rpo6 basic forms. Sequences are denoted by their numeric Genbank Identifiers (GI numbers) and phylum name. The alignment was constructed using the program MUSCLE 3.7 [75]. Similar residues are colored as the most conserved one (according to BLOSUM62): maximum score (3.0)—blue, minimum score (0.5)—grey. C-terminal β-finger sequences are marked with a red rectangle.

Archaea of the Iainarchaeota phylum also have two forms of Rpo6: basic and shortened. The first form contains the minimum number of amino acids (67–68 aa) necessary to preserve the tertiary structure of the basic form of Rpo6. Other representatives of archaea do not have such minimal basic forms of Rpo6 (Figure 10). The second form is similar to the shortened form of Euryarchaeota. The primary sequence of the basic forms of Rpo6 in Aenigmarchaeota, Undinaarchaeota, Naiadarchaeota, and Iainarchaeota differs significantly from Rpo6 in other archaea in the C-terminal region of the β-finger (Figure 11), indicating an early divergence of these organisms from the common ancestor of archaea and/or a rapid evolution of the primary structure of the Rpo6 subunit.

In other phyla of Nanobdellati, RNA polymerase contains one or two Rpo6 forms, which are similar to the forms of this subunit in the Euryarchaeota superphylum.

**Figure 12:**
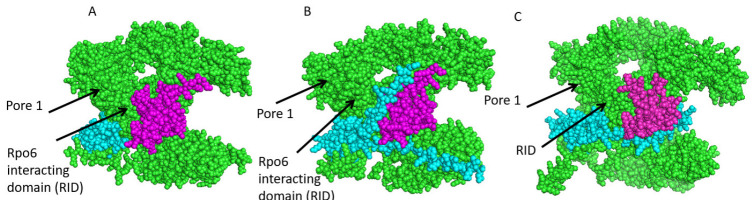
Three-dimensional interaction model of Rpo5 (magenta), Rpo6 (cyan) and the largest RNA polymerase subunit Rpo1 (green) predicted by AlphaFold3. (**A**) Euryarchaeota (*Thermococcus kodakarensis* (Rpo5: WP_011250035.1; Rpo6: WP_011250449.1; Rpo1: WP_011250033.1)); (**B**) Euryarchaeota (Methanocellales archaeon (Rpo5: MEM2924398.1; Rpo6: MEM2924456.1; Rpo1: MEM2924396.1, MEM2924395.1)); (**C**) Nanobdellati (Candidatus Woesearchaeota archaeon (Rpo5: PIN77523.1; Rpo6: PIN77917.1; Rpo1: PIN77526.1)). The confidence score of these models in all structured regions of the archaeal RNA polymerase is 70–90%.

The three-dimensional structural model of Rpo1-Rpo5-Rpo6 from *Thermococcus kodakarensis* showed that Rpo6 without the C-terminal region does not interact with RID and Rpo5, allowing Pore 1 and RID to vary (Figure 12A). The interaction model of the elongated form of Rpo6 (Methanocellales) reveals new contacts with RID and Rpo5, while Thermoproteati and Promethearchaeati do not have such contacts (Figure 12B). The elongated form of Rpo6 from Woesearchaeota forms new specific contacts with Rpo5 (Figure 12C).

#### 2.2.5. Eukaryotes

**Figure 13:**
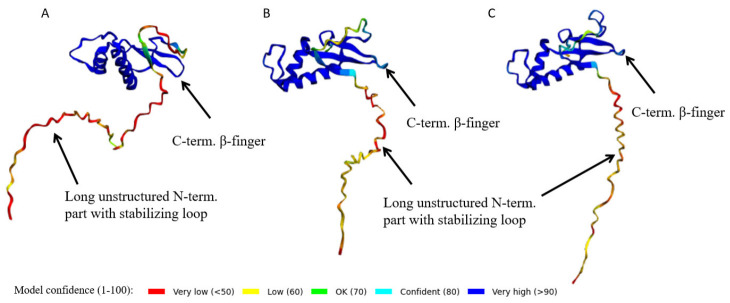
Three-dimensional structure of Rpb6 (Eucarya) predicted by AlphaFold3: (**A**) CAA18992.1 Rpb6 [*Schizosaccharomyces pombe*]; (**B**) NP_068809.1 Rpb6 [*Homo sapiens*]; (**C**) NP_524910.1 Rpb6 [*Drosophila melanogaster*].

In all eukaryotes, the Rpb6 subunit (a homolog of Rpo6) has an elongated shape and contains a large number of negatively charged amino acids at the N-terminus. The N-terminal region of this eukaryotic subunit has a more complex three-dimensional structure than the N-terminal sequences of the elongated forms of Rpo6 in archaea (Figure 13). This can be attributed to the emergence of new functions in the N-terminal region of eukaryotic Rpb6. For example, it was recently discovered that the N-terminal part of the human Rpb6 protein interacts with the p62 subunit of the general transcription factor TFIIH [42].

#### 2.2.6. Spearman’s Test for Correlation

To confirm our hypothesis about the loss of Rpo8, we performed a correlation analysis between the presence/absence of the Rpo8 subunit and the number of Rpo6 forms in different archaeal phyla. The Spearman’s test revealed a notable negative correlation (ρ = −0.579; *p* < 0.001). This means that the absence of Rpo8 correlates with an increase in the number of different Rpo6 forms.

#### 2.2.7. Phylogenetic Analysis

To determine the most ancient form of Rpo6, we performed a phylogenetic analysis of all the above-described forms of Rpo6.

**Figure 14:**
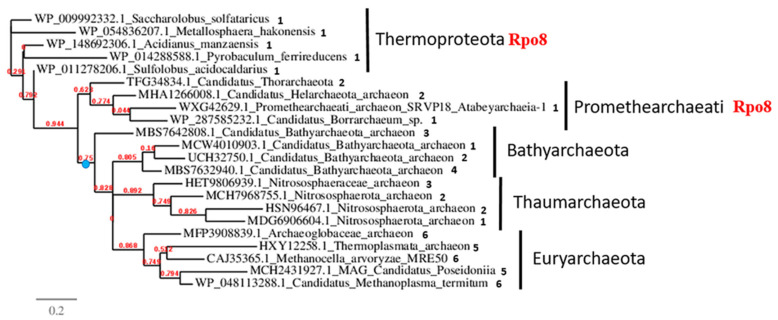
The phylogenetic tree based on the amino acid sequences of Rpo6 forms including representatives of Thermoproteota, Promethearchaeati, Bathyarchaeota, Thaumarchaeota and Euryarchaeota (Rpo6 forms of Nanobdellatti were not used due to the LBA effect). The maximum likelihood phylogenetic tree of Rpb6 forms was constructed by using the PhyML program and rendered by the program TreeDyn. Probabilities are indicated for selected major branches in red. Each terminal node of the tree is labeled by GI number, name of organism and Rpo6 form number (Table 1). The blue dot indicates the site of loss of the Rpo8 subunit.

The resulting phylogenetic tree shows that the ancestor of the Rpo6 Thetmoproteota forms is most ancient (Figure 14). The ancestor of Rpo6 Promethearchaeati and the common ancestor of Rpo6 Bathyarchaeota, Thaumarchaeota and Euryarchaeota diverged from it. Apparently, after this branching, the common ancestor of Bathyarchaeota, Thaumarchaeota, and Euryarchaeota lost the Rpo8 subunit, which led to the formation of new Rpo6 forms (from 2 to 6, see Table 1).

### 2.3. Limitations of Comparative Protein Modeling Methods

Comparative protein structure modeling has a number of limitations related to the specific methods, biological factors, and computational constraints.

1. Sequence alignment and template. Model quality depends on the quality of sequence alignment and template selection. At low sequence identity (e.g., below 20%), the structure may differ significantly from the expected one.
2. Conformational flexibility of proteins. Proteins often have several functionally significant states. A static conformation does not fully represent the dynamic properties that determine catalytic activity, allosteric regulation, and interactions with ligands.
3. Limitations of machine learning methods. The quality of predictions depends on the volume and representativeness of the training data. Deep learning algorithms like AlphaFold require large, high-quality datasets for training.
4. Machine learning methods often ignore obvious physical limitations or take them into account in a simplified form. These assumptions can lead to the formation of structures that formally conform to the statistical patterns of the training data, but contradict certain physical properties.

The training dataset for AlphaFold3 (v3.0.1) contains all structures released in the PDB before 30 September 2021. This dataset contains more than 50 three-dimensional structures of archaeal RNA polymerase and eukaryotic RNA polymerase II (that most similar to archaeal enzyme) with high resolution. In addition, proteins of the transcription machinery and especially subunits of RNA polymerases show significant homology between related proteins in different organisms. This gives confidence that the protein models for RNA polymerase subunits predicted using AlphaFold3 have significant accuracy.

## 3. Discussion

We traced changes in the primary and domain structures of two small RNA polymerase subunits (Rpo6 and Rpo8) in all known archaea phyla to date and compared them with homologous eukaryotic proteins. We found that the domain structure of Rpo6 varies greatly in different archaea types. This is surprising, since the structure of Rpb6 (its C-terminal part) is very conserved in eukaryotes [38,39,40]. Our analysis of the primary and domain structure of Rpo6 in different archaeal phyla showed that the largest number of significant changes in this small subunit occurred in archaea with RNA polymerase without the Rpo8 subunit. Shortened forms of Rpo6 without N- and C-terminal regions were observed only in archaea with RNA polymerase that does not contain the Rpo8 subunit.

Changes in interactions in the three-dimensional models of the Rpo1-Rpo5-Rpo6 module of some archaea are observed if they do not have the Rpo8 subunit and, at the same time, contain shortened or elongated forms of Rpb6. It is likely that such changes are caused by the disappearance of the Rpo8 subunit from the RNA polymerase complex of archaea.

Our results show that the Rpo8 subunit was apparently present in the RNA polymerase of the last common ancestor of the archaea (LCAA) and disappeared during evolution in the superphyla Euryarchaeota, Nanobdellati, and the phyla Bathyarchaeota and Thaumarchaeota (superphylum Thermoproteati).

Other scenarios for the evolution of Rpo8 are less plausible. One of them suggests the transfer of the Rpo8 gene to Thermoproteota (Thermoproteati) and Promethearchaeati from eukaryotes. Another is the occurrence of Rpo8 de novo or a change in the cellular functions of some other protein binding RNA/polysaccharides (for example, ribosomal protein) in these taxa. Under such evolutionary scenarios, the variability of the Pore 1 domain would be high only in Thermoproteota and Promethearchaeati, since the integration of Rpo8 into RNA polymerase would require some changes in this domain. In other superphyla, the variability of this domain would be significantly lower. Comparison of archaeal Pore 1 domains (see Table 2) indicates that the representatives of all superphyla have high variability (low homology) of these domains.

It can also be assumed that the most ancient form of Rpo6 is a subunit in representatives of Thermoproteota, which have a single variant (70–120 amino acids long). The same form was found in the phyla Bathyarchaeota and Thaumarchaeota, as well as in the superphyla Promethearchaeati and Nanobdellati. This assumption was confirmed by the result of phylogenetic analysis (Figure 14).

In order for archaea to evolve and adapt to new habitats and food sources, changes in the structure of RNA polymerase were necessary, particularly in the area of interaction with transcription factors. Since the Rpo6 subunit is the primary area of interaction with the transcription initiation factor TFE, changes in its structure (such as shortening, lengthening, etc.) have contributed to more precise regulation of the transcription process. However, this also required changes in the Rpo8 subunit. The changes of RNA polymerase in archaea have occurred in two distinct directions. The first is the disappearance of the Rpo8 subunit from RNA polymerase (in Bathyarchaeota, Thaumarchaeota, Nanobdellati, and Euryarchaeota), and the second is a radical change in the primary structure of Rpo8, which appears to have provided a more flexible interaction with the enzyme complex and allowed the Rpo6 subunit to change (in Promethearchaeota). In addition, it turned out that Rpo6 underwent characteristic changes during evolution in all types of archaea, which can undoubtedly be used for their classification.

## 4. Materials and Methods

To identify candidate RNA polymerase Rpb8 and Rpb6 subunits in archaea, a set of coding sequences (from Thermoproteota and Eukarya) was individually used as queries to search the nr cluster database of the National Center for Biotechnology Information (NCBI, Bethesda, MD, USA, https://blast.ncbi.nlm.nih.gov/ (accessed on 21 March 2026)) using the PSI-BLAST (online version) algorithm (non-redundant proteins grouped by 90% identity and 90% coverage). The coding sequences of Eukarya and Thermoproteota were obtained from the Protein Database and Reference Sequence (RefSeq) database of the National Center for Biotechnology Information (https://www.ncbi.nlm.nih.gov/guide/proteins/ (accessed on 21 March 2026)). Multiple alignment of the amino acid sequences of the Rpo8 and Rpo6 proteins of archaea was performed using the MUSCLE 3.7 program [76]. The three-dimensional structures of Rpo8 and Rpo6 proteins were predicted using AlphaFold3 (https://alphafoldserver.com/ (accessed on 21 March 2026)) [77]. Figures with models of Rpo1-Rpo5-Rpo6-Rpo8 and/or Rpo1-Rpo5-Rpo6 interactions were prepared using the PyMOL molecular graphics system (version 3.1.7.2.). The Spearman’s test was applied using R 4.3.3., the “stat” package (R Core Team, 2024) in the RStudio 2024.12.0 integrated development environment (Posit Software, PBC, Boston, MA, USA). The phylogenetic analysis was performed using the PhyML 3.0 [78] and visualized using the TreeDyn 198.3 programs [79].

## Figures and Tables

**Table 1:** Different forms of the RNA polymerase Rpo6 subunit from different archaeal superphyla. The green rectangles represent central α-helices, the blue rectangle—C-terminal β-finger, the red rectangle—the stabilizing loop.

N	Rpo6 Form	Form Description	Presence in Archaea Phyla
1	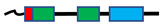	Basic form: shot N-terminus, stabilizing loop, two central α-helices, C-terminal β-finger	Thermoproteota, Promethearchaeati, Bathyarchaeota, Thaumarchaeota, Nanobdellati
2	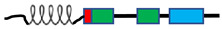	Long structured N-terminus, stabilizing loop, two central α-helices, C-terminal β-finger	Thaumarchaeota, Bathyarchaeota
3	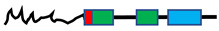	Long unstructured N-terminus, stabilizing loop, two central α-helices, C-terminal β-finger	Promethearchaeati, Bathyarchaeota, Thaumarchaeota
4	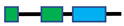	Truncated N-terminus, two central α-helices, C-terminal β-finger	Bathyarchaeota
5	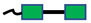	Shot N-terminus, two central α-helices, truncated C-terminus	Euryarchaeota, Nanobdellati
6	** 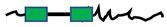 **	Shot N-terminus, two central α-helices, unstructured C-terminus	Euryarchaeota, Nanobdellati

**Table 2:** The average percentage of identical amino acids in the Pore1 domain (Rpo1) across representatives of various archaeal taxa. Randomly selected sequences of the Pore1 domain from representatives of Thermoproteota, Promethearchaeati, Bathyarcheota, Thaumarcheota, Euryarchaeota, and Nanobdellati were compared with homologous proteins from aforementioned taxa using the Blast program. The average percentage of identical amino acids was calculated using a set of 500 proteins.

	Thermo-Proteota	Promethe-Archaeati	Bathy-Archeota	Thaum-Archeota	Eury- Archeota	Nano- Bdellatti
**Thermoproteota** WP_338599920.1 Pore1 domain Rpo1 (491-620 a.a.)	–	39.9	45.7	42.6	36.9	36.0
**Promethearchaeati** WP_445870884.1 Pore1 domain Rpo1 (505-622 a.a.)	44.9	–	44.4	42.5	35.6	33.7
**Bathyarchaeota** MHD7586758.1 Pore1 domain Rpo1 (491-625 a.a.)	41.5	41.9	–	47.6	42.0	34.4
**Thaumarcheota** HET9612740.1 Pore1 domain Rpo1 (495-618 a.a.)	38.6	38.9	45.6	–	34.8	34.5
**Euryarchaeota** WP_457549491.1 Pore1 domain Rpo1 (962-1105 a.a.)	41.3	42.8	44.9	38.1	–	35.1
**Nanobdellati** RME78117.1 Pore1 domain Rpo1 (481-610 a.a.)	30.5	30.1	29.2	33.4	32.0	–

## Data Availability

The original contributions presented in this study are included in the article. Further inquiries can be directed to the corresponding author(s).

## Abbreviations

TFB	archaeal transcription factor B
TFE	archaeal transcription factor E
TFIIB	general transcription factor B of RNA polymerase II
TFIID	general transcription factor D of RNA polymerase II
TFIIE	general transcription factor E of RNA polymerase II
TFIIF	general transcription factor F of RNA polymerase II
TFIIH	general transcription factor B of RNA polymerase II
TFIIS	eukaryotic transcription elongation factor S of RNA polymerase II
RID	Rpo6 interacting domain
